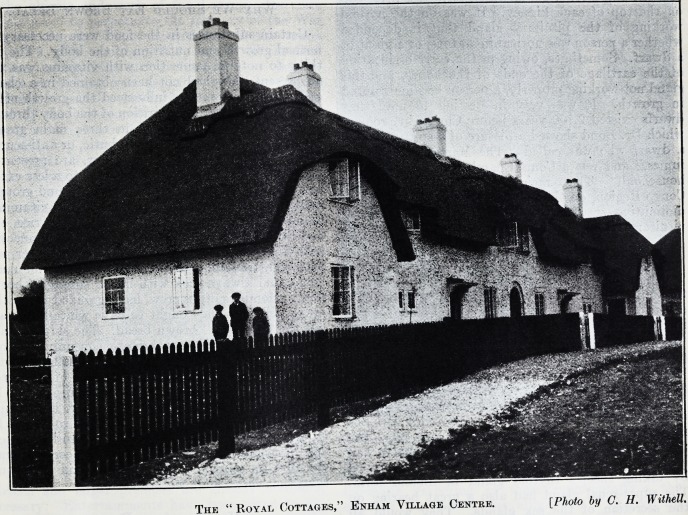# Enham Village Centre

**Published:** 1924-06

**Authors:** 


### ENHAM VILLAGE CENTRE.

The Enham Village Centre, Andover, where a valuable work is being accomplished in the training of ex-service men and the provision of permanent settlement for them, was visited recently by Princess Mary and Viscount Lascelles, who declared open four Royal cottages in commemoration of a visit paid in 1922 by the King and Queen and the Duke of York. The cottages were built with the money (£4,000) allocated by Princess Mary and her husband from their Wedding Present Fund. Her Royal Highness presented to Mr. George Livermore the certificate awarded to him by the Carnegie Hero Trust Fund for heroism. During a fire in a hut Mr. Livermore at great risk carried a blazing bucket of beeswax and turpentine outside, and, although he saved the hut and his fellows, he lost part of his left arm and received terrible burns.

Enham Place, the medical block, was inspected and a tour was made of the factory, where Princess Mary was asked to accept some Enham products—a basket, a pound of butter and some honey. She was much interested in the smallholder, the settlers and the garden trainees, and in a subsequent speech Lord Lascelles said that they had selected Enham for their gift, feeling sure, from what they had heard of its management, that no place would spend the money to better advantage. The photograph we give was taken by Mr. C. H. Withell, one of the disabled men.

The "Royal Cottages," Enham Village Centre. [*Photo by C. H. Withell.*

## Figures and Tables

**Figure f1:**